# Efficacy of Acupuncture and Moxibustion as a Subsequent Treatment after Second-Line Chemotherapy in Advanced Gastric Cancer

**DOI:** 10.1155/2020/8274021

**Published:** 2020-06-29

**Authors:** Yong-jie Zhang, Qi Min, Ying Huang, Huai-dong Liu, Zi-yuan Zhu, Fu-jin Jiang, Hai-qing Hua

**Affiliations:** ^1^Huai'an Hospital Affiliated to Xuzhou Medical University, Huai'an 223002, Jiangsu, China; ^2^General Hospital of Eastern Theater Command Affiliated to Nanjing University of Chinese Medicine, Nanjing 210002, Jiangsu, China

## Abstract

**Objective:**

To explore whether acupuncture and moxibustion can prevent disease progression of advanced gastric cancer patients completing second-line chemotherapy and, if so, the related mechanism.

**Method:**

Progression-free survival (PFS) and overall survival (OS) were main outcome measures. The real-time quantitative PCR was used to detect the expression of genes including T-bet, IFN-*γ*, GATA3, and IL-4 in peripheral blood mononuclear cells (PBMCs). IL-4, IL-6, Ca199, CRP, and IFN-*γ* in plasma levels were checked.

**Results:**

170 patients were randomly assigned in a 3 : 2 ratio to receive either acupuncture and moxibustion or sham acupuncture until progression. 135 patients were included in the primary analysis. Both PFS and OS in treatment group were proven to be better than control group. Acupuncture and moxibustion promoted typical Th1 cells drifting, as confirmed by increased T-bet/IFN-*γ* and decreased GATA3/IL-4 in mRNA levels from PBMCs, as well as upregulating IFN-*γ* and downregulating IL-4 in plasma levels. IL-6, Ca199, and CRP in plasma levels were also reduced by acupuncture and moxibustion.

**Conclusions:**

Acupuncture and moxibustion can prolong PFS and OS of advanced gastric cancer patients completing second-line chemotherapy by reversing Th1/Th2 shift and attenuating inflammatory responses.

## 1. Introduction

Gastric cancer, characteristic of high incidence, metastasis rate, and mortality rate, ranks as the 5th commonest cancer and the 3rd leading reason for cancer-associated mortality worldwide. Although the precise mechanism of gastric cancer progression remained to be elucidated, the causal effect of the switch of immune state has recently attracted increasing attention. T helper type 1 (Th1) as well as Th2 and T17 cells were identified as subtypes of helper T cells. Th1 cells, arising from interleukin-12 (IL-12) stimulation, create interleukin-2 (IL-2) and interferon gamma (IFN-*γ*) and regulate cytotoxic T lymphocyte (CTL) response mediating antitumor effects. In contrast, Th2 cells, arising from IL-4 stimulation, create IL-4 and IL-10 and favor tumor growth by suppressing the generation of Th1 and CTLs [1, 2]. Th17 cells, arising from stimulation of transforming growth factor (TGF)-*β* and IL-6, play a significant part in inflammation and tumor development [3].The coprecursor cell of Th1 and Th2 is Th0 cells. T-bet and GATA3 are responsible for regulating the differentiation of Th0 into Th1 and Th2, respectively. Weak T-bet expression and/or strong GATA3 expression accounts for predominant Th2 type cytokines in gastric cancer patients [4]. Th1 and Th2 cells, as well as their associated cytokines, coordinate the delicately organized events that govern steady state of immune system by cross-inhibiting each other [1], resembling Yang and Yin in Traditional Chinese Medicine (TCM), which are mutually interdependent and conditioned. The dialectical unity of Yang and Yin forms the philosophical basis of TCM [5], keeping the overall balance of the living system. Deficiency of Yang or excess of Yin can upset the homeostasis of the whole system, leading to all sorts of pathological changes including formation and growth of tumor. Impaired cell-mediated immunity of cancer patients is presented as switch from Th1 to Th2, namely, transition from Yang to Yin from the perspectives of TCM. Therefore, successful treatment strategies to rebalance Th1/Th2 by promoting Th1 dominance can reduce the risk of cancer relapse or progression.

Acupuncture, an important part of TCM, has been accepted and used by at least 103 countries according to the WHO statistics due to favorable clinic effect and minor side effects [6]. Considerable research focused on the role of acupuncture in improving cancer-related symptoms including pain, anxiety, fatigue, hot flushes, depression, shortness of breath, insomnia, and gastrointestinal side effects [7]. Further research indicated that acupuncture can improve inflammation by decreasing proinflammatory cytokine IL-6 and Th2 cytokines including IL-4 and IL-10, as well as by increasing Th1 cytokines such as IL-2 and IFN-*γ* [8]. Moxibustion, similar to acupuncture therapy in terms of the meridian system and acupoint theories, is a noninvasive procedure that involves burning moxa, the herb *Artemisia vulgaris*, on or above the skin at acupoints, bringing warmth back to the Yang and eliminate cold in the Yin. Moxibustion can reduce inflammatory response by decreasing proinflammatory cytokine IL-6 [9, 10]. Considering the decisive role of Th1/Th2 imbalance and inflammatory responses in tumor promotion, it is tempting to speculate that acupuncture and moxibustion could suppress tumor development by reversing the imbalance of Th1/Th2 and inhibiting inflammation. As far as we know, this is the first study to describe the effects of acupuncture and moxibustion on tumor inhibition.

In the current study, we explored whether progression-free survival (PFS) and overall survival (OS) can be prolonged by acupuncture and moxibustion treatment of advanced gastric cancer patients completing second-line chemotherapy. Furthermore, the correlation of acupuncture and moxibustion with T-bet/IFN-*γ*, GATA3/IL4, inflammation markers IL-6 and C-reactive protein (CRP), and tumor marker Ca199 was evaluated. Our results demonstrate that acupuncture and moxibustion contributes to inhibiting progression and improving prognosis of advanced gastric cancer by reversing Th1/Th2 shift and downregulating IL-6, CRP, and Ca199.

## 2. Methods

### 2.1. Participants

An assessor-blinded, randomized, controlled study was conducted in patients with locally advanced, metastatic, or recurrent gastric adenocarcinoma. In order to be included, participants had to fulfill all the following criteria: (1) ≥18 years old; (2) histologically confirmed diagnosis; (3) completion of second-line chemotherapy; (4) Eastern Cooperative Oncology Group (ECOG) performance status 0-1; (5) at least one measurable lesion; (6) informed consent. Participants were excluded if they had any of the following: (1) a past history of checkpoint inhibitor-based immunotherapies; (2) severe cardiovascular, hepatic, or renal disease; (3) current infection; (4) acupuncture or moxibustion treatment within 1 month before the trial; (5) autoimmune disease; (6) immunosuppressive agents or hormone therapy in the past 6 months. The eligible participants were then randomly assigned in 3 : 2 ratio to receive acupuncture and moxibustion (treatment group) or sham acupuncture (control group). Randomization was performed by the School of Public Health of Nanjing Medical University. Neither study personnel nor patients were masked to treatment assignment. The independent assessor evaluating outcome had no knowledge of treatment allocation. This study was performed based on the common guidelines for clinical trials in line with the Declaration of Helsinki. The study was retrospectively approved by review board and ethics committee of Huai'an Hospital Affiliated with Xuzhou Medical University on 30 November 2016 (reference no. HEYLL201605).

### 2.2. Interventions

Patients in treatment group received acupuncture performed at Zusanli (ST36), Yuji (LU10), Tianxi (SP18), Pigen (EX-B4), Diwuhui (GB42), and Yingchuang (ST16).

The acupuncture needle was inserted vertically at ST36, LU10, and EX-B4 and obliquely at SP18 and ST16 with arrival of qi [11], referring to the sensation of soreness, numbness, swelling, and pain. Moxibustion was performed following acupuncture. The acupoints of moxibustion therapy consisted of Zhongwan (RN12), Zusanli (ST36), Weishu (BL21), Shenshu (BL23), and Gaohuang (BL43). Dried mugwort in a cone shape (1.8 cm in basal diameter and 2.0 cm in height; Henan Nanyang Medical Co., Ltd., China) was placed on moxibustion acupoints and then was lit and removed after it burned two-thirds of its height. The course was continuously repeated 3 times on every acupoint. Patients in control group were treated with superficial needle puncture at nonacupoint sites, located 2 cm away from the acupoints of the treatment group. The needling depths were too superficial to obtain arrival of qi, minimizing acupuncture-induced physiologic effects. The needle-retaining time in treatment and control groups was 30 min. The size of acupuncture needles is 0.25 *∗* 50 mm (Suzhou Dongbang Medical Co., Ltd., China). All interventions were given until disease progression according to Response Evaluation Criteria in Solid Tumors, version 1.1 (RECIST 1.1).

### 2.3. Blood Samples

We collected peripheral blood samples from all patients. After being acquired by standard Ficoll-Hypaque density centrifugation, peripheral blood mononuclear cells (PBMCs) were kept in storage at -70◦C for detecting transcription factors and cytokines.

### 2.4. Design of Primers and Response Conditions

The genetic testing primers were designed by Premier 5.0 software based on GenBank sequences and then synthesized by Shanghai Sangon Biological Engineering Technology and Services Co., Ltd. Primer's sequences are displayed in Table 1.

### 2.5. RNA Extraction and cDNA Synthesis

We extracted total RNA from PBMCs using Trizol (Invitrogen, USA) and synthesized cDNA with reverse transcription reagent kits (TOYOBO, Japan) following the manufacturer's instructions.

### 2.6. The Real-Time Quantitative PCR for Detecting Genes Expression

We detected the expression of genes including T-bet, IFN-*γ*, GATA3, and IL-4 by real-time quantitative PCR and calibrated all samples by GAPDH. We calculated the concentration of T-bet, IFN-*γ*, GATA3, and IL-4 transcripts in samples with the Corbett software and then obtained the expression levels of T-bet, IFN-*γ*, GATA3, and IL-4 based on each standard curve. The expression of T-bet, IFN-*γ*, GATA3, and IL-4 was normalized against that of GAPDH. We checked all samples in triplicate.

### 2.7. Objectives and Definitions

The purpose of our study was to compare PFS and OS between treatment and control groups. PFS was measured as the time since enrollment until the recorded cancer recurrence or death due to any cause. OS was calculated from the time of enrollment to death from any cause. Patients were followed up until death, loss to follow-up, or end of study. The follow-up deadline was December 2019.

### 2.8. Statistical Analysis

The quantitative data was analyzed by Chi-squared test. Quantitative data were exhibited as mean ± standard deviation (SD). Appropriate Student's *t*-test was used to compare data of mRNA levels. The data of plasma levels was evaluated by Mann–Whitney *U* test because of nonnormal distribution. Pearson rank test was applied to explore correlation between two continuous variables. The Kaplan–Meier method and log-rank test were used to assess survival rate and the difference of survival rates, respectively. *P* < 0.05 was regarded as being statistically significant. We performed all statistical analysis using SPSS16.0 software (SPSS Inc., Chicago, IL, USA).

## 3. Results

**Figure 1:**
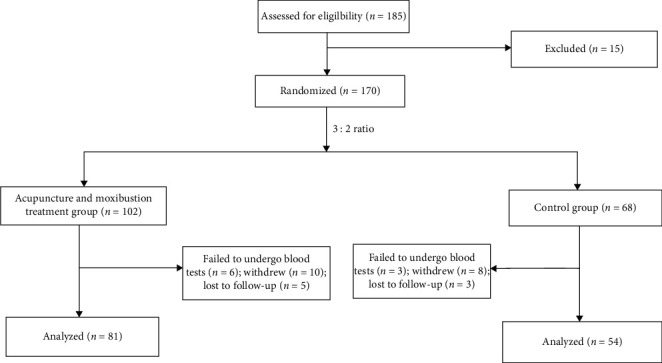
Low chart of participants.

From October 2015 to March 2019, 185 patients with advanced gastric cancer completing second-line chemotherapy attending our clinic were screened. According to inclusion and exclusion criterion, 170 eligible patients signed the informed consent form and were randomly assigned in a 3 : 2 ratio (102 in treatment group; 68 in control group). Nine patients failed to undergo blood tests at week 4 or 8 (six in treatment group; three in control group). Eighteen patients withdrew from the study for personal reasons (ten in treatment group; eight in control group) and eight patients failed to be followed up (five in treatment group; three in control group). Therefore, a total of 135 patients were included in the final analysis, 81 in treatment group and 54 in control group (Figure 1).

### 3.1. Long-Term PFS and OS

**Figure 2:**
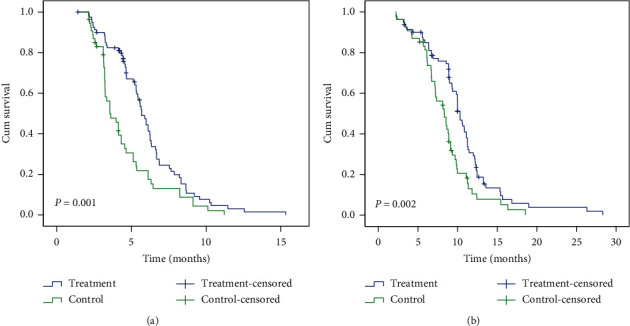
Both progression-free survival (PFS) (a) and overall survival (OS) (b) in treatment group were significantly better than control group.

The baseline data for patients with advanced gastric cancer in treatment and control groups were comparable in terms of age, sex, ECOG performance status, location and size of primary tumor, differentiation, metastatic sites per patient, extent of disease at study entry, and type of gastric cancer (Table 2). Third-line therapy after disease progression was given to 35 patients (43%) in treatment group (16 patients [20%] received chemotherapy and 19 patients [23%] received tyrosine kinase inhibitor apatinib) [12] and 25 patients (49%) in control group (12 patients [24%] received chemotherapy and 13 patients [25%] received apatinib). Median follow-up was 15.61 months in treatment group and 14.32 months in control group. PFS was significantly longer with acupuncture and moxibustion than with sham acupuncture (median, 5.68 months [95% CI, 5.22 to 6.14] vs. 3.56 months [95% CI, 2.82 to 4.30]; *P*=0.001) (Figure 2(a)). OS was dramatically prolonged in acupuncture-moxibustion group compared with sham acupuncture group (median, 10.32 months [95% CI, 9.73 to 10.92] vs. 8.35 months [ 95% CI, 6.98 to 9.72]; *P*=0.002) (Figure 2(b)).

### 3.2. PCR Identified by Electrophoresis

The sequences of genes including T-bet, IFN-*γ*, GATA3, IL-4, and GAPDH were identical to the ones offered by the National Center of Bioinformatics Institute (NCBI). The length of the amplified target fragment of T-bet, IFN-*γ*, GATA3, IL-4, and GAPDH was 279, 388, 339, 368, and 205 bp, respectively, which conformed to the expected ones (Table 1).

### 3.3. Effect of Acupuncture and Moxibustion on mRNAs of T-bet, IFN-*γ*, GATA3, and IL-4 in PBMCs

**Figure 3:**
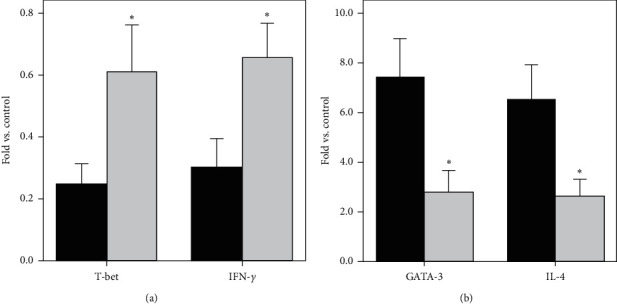
Effect of acupuncture and moxibustion on mRNA expression of transcription factors and cytokines in PBMC. (a) The mRNA expression of T-bet and IFN-*γ* increased obviously at week 4 (grey) compared with baseline (black). (b) The mRNA expression of GATA3 and IL-4 decreased remarkably at week 4 (grey) compared with that at baseline (black). ^*∗*^*P* < 0.01 (Student's *t*-test).

The mRNA expression of T-bet, IFN-*γ*, GATA3, and IL-4 in treatment group was measured by the real-time quantitative PCR at week 0 (baseline) and week 4. The mRNAs of T-bet and IFN-*γ* increased from 0.25 ± 0.07 and 0.30 ± 0.09 at baseline to 0.61 ± 0.15 (*P* < 0.001) and 0.66 ± 0.11 (*P* < 0.001) at week 4, respectively (Figure 3(a)), while the mRNAs of GATA3 and IL-4 dramatically decreased from 7.47 ± 1.51 and 6.50 ± 1.43 at baseline to 2.77 ± 0.88 (*P* < 0.001) and 2.63 ± 0.69 (*P* < 0.001) at week 4, respectively (Figure 3(b)). Our data indicated that acupuncture and moxibustion promotes a typical Th1 cells drifting by upregulating T-bet/IFN-*γ* and downregulating GATA3/IL-4, enhancing immune inhibition of advanced gastric cancer which originally exhibits predominant expression of Th2 type cytokines. We further testified the results in plasma levels. Considering the importance of immunity in controlling tumor progression, we studied whether acupuncture and moxibustion can decrease gastric cancer marker Ca199 and proinflammatory factors IL-6 and CRP, which were intimately related with development of gastric cancer.

### 3.4. Effect of Acupuncture and Moxibustion on IFN-*γ*, IL-4, IL-6, Ca199, and CRP in Plasma

We examined IFN-*γ*, IL-4, and IL-6 in plasma levels in treatment and control groups at baseline and weeks 4 and 8. There is no statistical significance between treatment and control groups at baseline plasma levels of IFN-*γ* [(3.90 ± 1.82 vs. 3.93 ± 0.75) pg/ml; *P* > 0.05], IL-4 [(3.29 ± 1.04 vs. 3.48 ± 1.10) pg/ml; *P* > 0.05], and IL-6 [(79.83 ± 41.72 vs. 81.79 ± 25.67) pg/ml; *P* > 0.05]. In treatment group, IFN-*γ* levels increased from baseline to 5.88 ± 1.56 pg/ml (*P* < 0.01) at week 4 and 5.81 ± 1.45 pg/ml (*P* < 0.01) at week 8. IL-4 levels decreased from baseline to 1.82 ± 0.55 pg/ml (*P* < 0.01) at week 4 and 1.65 ± 0.42 pg/ml (*P* < 0.01) at week 8. Change trends of IL-4 and IFN-*γ* in plasma levels were entirely consistent with those in mRNA levels from PBMCs. IL-6 levels decreased from baseline to 53.29 ± 29.50 pg/ml (*P* < 0.01) at week 4 and 51.18 ± 31.98 pg/ml (*P* < 0.01) at week 8. There is no statistical significance between treatment and control groups at baseline plasma levels of CRP [(39.73 ± 16.20 vs. 36.52 ± 14.30) U/mL; *P* > 0.05] and Ca199 [(60.53 ± 34.21 vs. 58.57 ± 22.57) U/mL; *P* > 0.05]. In treatment group, CRP levels decreased from baseline to 28.11 ± 15.63 mg/L (*P* < 0.01) at week 4 and 30.32 ± 12.53 mg/L (*P* < 0.01) at week 8. Ca199 levels decreased from baseline to 45.83 ± 21.31 U/mL (*P* < 0.01) at week 4 and 43.59 ± 25.03 U/mL (*P* < 0.01) at week 8. However, the change trends of IL-4, IFN-*γ*, IL-6, CRP, and Ca199 at weeks 4 and 8 compared with at baseline in control group were opposed to those in treatment group, which contributed to more significant statistical differences of IL-4, IFN-*γ*, IL-6, CRP, and Ca199 in plasma levels between treatment and control groups (Table 3). Altogether, our results showed that acupuncture and moxibustion can inhibit gastric cancer development by promoting Th1 dominance and decreasing tumor marker Ca199 and proinflammatory factors IL-6 and CRP.

## 4. Discussion

Based on the important role of inflammation at various stages of tumor development, from initiation to local invasion and distant metastasis, tumors are known as “wounds that do not heal” [13, 14]. Inflammation involves interaction between immune cells, cytokines, chemokines, inflammatory cells, and proinflammatory mediators. During this process, the immune cells and inflammatory cells are “educated” by the tumor to acquire tumor-promoting activities, which simultaneously provides encouraging therapy strategies achieved by anti-education. Thus, illustrating the core factors playing critical role in inflammation will ultimately promote the development of therapeutic approaches with durable antitumor responses.

Gastric cancer presents with high heterogeneity and most of various target-specific therapies for patients in advanced stages were unsuccessful in controlled clinical trials, challenging formulation of rational treatment strategies. The inflammation-driven trait of gastric cancer may provide an opportunity of replying the challenge. There exists close association of *H. pylori* infection with the oncogenesis of gastric cancer. *H. pylori* can activate and enhance cytokine signaling such as IL-8 and TNF-alpha by stimulating nuclear factor kappa B (NF-*κ*B), an essential inflammatory regulator. Another important inflammatory regulator, STAT3, greatly promotes tumor growth and is referred to as a hallmark of gastric cancer [15]. The interplay between TLR/MyD88 and COX-2/PGE2 pathways also contributes to inflammatory response of gastric cancer [16]. Of the tumor-infiltrating lymphocytes, Th lymphocytes, acting as markers of inflammation response and main players in tumor immunity, play an important role in tumor progression [17]. T-bet induces Th1 cytokine IFN-*γ* and reduces Th2 cytokine IL-4, while GATA-3 promotes the Th2 response and inhibits Th1 differentiation. IL-4 produced by Th2 cells decreases IFN-*γ* secretion [18]. The cross regulation and interrestraint between Th1 and Th2 cells sustain the balance of immune system. Tumor progression resulted from inability of immune system to maintain tumor-destructive Th1 state and switching to Th2 dominance in many kinds of tumor including gastric cancer [5]. Sustained immunologic shift from Th2 to Th1 is considered to be essential for curative treatment. Many high-quality studies have demonstrated that IFN-*γ* -producing Th1 cells make great contributions to antitumor responses [19–22], and their infiltration always indicates better prognosis [23]. The proportion of Th2 cells in tumor tissue of advanced hepatocellular carcinoma, whether before or after intra-arterial chemotherapy, was remarkably lower in patients with partial response or stable disease than in patients with progression [24]. One month after tumor excision, lymphocytes producing Th2 were also downregulated considerably. All of these studies disclosed the important role of the shift from Th2 to Th1 in successful treatment of tumor.

IL-6 is a versatile cytokine responsible for regulating both immune and inflammatory responses and acts as an important modulator of activity of immune cells associated with tumor development. IL-6 can not only expand myeloid derived suppressor cells (MDSC), but also activate Th17 cell response and inhibit the differentiation of dendritic cells. IL-6 can impair Th1 differentiation through stimulating IL-4/IL-21 production and therefore has negative impacts on antitumor responses by shifting Th1/Th2 balance to Th2 dominance [21], leading to unfavorable prognosis in a wide range of tumor types including gastric cancer. Furthermore, IL-6 derived from tumor-associated fibroblasts infiltrating into gastric cancer microenvironment can promote epithelial-mesenchymal transition (EMT) [25]. Thus, IL-6-targeting therapy can be taken advantage of to inhibit gastric cancer.

Acupuncture, as an important part of TCM, has served Chinese people for five thousand years. Scientific evidence and remarkable clinical efficacy have made acupuncture accepted worldwide and brought into being Western Medical Acupuncture. Acupuncture is based on the theory that stimulating certain particular acupressure on the body with needles can rebalance Yin and Yang by regulating the flow of “Qi,” or vital energy. Scientific evidence demonstrates that acupuncture can result in releasing a great variety of neurotransmitters and changes in MRI signals of brain function, indicating the mediating effects of nervous system during acupuncture. Acupuncture treats acute pancreatitis by downregulating NF-*κ*B and proinflammatory cytokines [26]. Electroacupuncture also has therapeutic effect on acute pancreatitis by decreasing IL-6, leading to reducing the inflammatory response [27–29]. Acupuncture dramatically reduces IL-4 and IL-10 and upregulates IFN-*γ* in allergic rhinitis patients, causing a swift of Th1/Th2 balance towards Th1 [30–32]. Acupuncture can remarkably decrease IL-6 and IL-10 in asthma patients [33]. Compared with control and sham acupuncture groups, electroacupuncture upregulates IFN-*γ* and downregulates IL-4 and IL-10 in bronchoalveolar lavage and pulmonary tissue in asthma rodent model [34]. All of these studies disclose that acupuncture can promote rehabilitation of inflammatory disease by shifting Th1/Th2 balance to Th1 dominance and downregulating proinflammatory cytokine IL-6. Moxibustion can regulate the immune function and reverse Th1/Th2 cytokine imbalance of athletes during course of heavy load training [35–37]. Our data showed that acupuncture combined with moxibustion can upregulate T-bet/IFN-*γ* and downregulate GATA3/IL-4 in mRNA levels from PBMCs and thus cause Th1/Th2 balance towards strongly favoring Th1 dominance, leading to favorable antitumor effect. Upregulation of IFN-*γ* and downregulation of IL-4 and IL-6 in plasma levels were also verified.

As an inflammation hallmark and direct target of IL-6 signaling, CRP is closely related to progression in a wide variety of cancers including gastric cancer [38]. CRP, Ca199, and IL-6 in plasma levels increase with the gastric wall invasion. Plasma concentrations of IL-6 and Ca199 are associated with lymph node metastasis and CRP with stage, size, lymph node, and distant metastases [39]. Our data indicated that Ca199, CRP, and IL-6 were dramatically downregulated by acupuncture and moxibustion. Both PFS and OS in treatment group were prolonged compared with control group. Considering the intimate association of Th1/Th2 imbalance and inflammatory factors including CRP and IL-6, as well as gastric cancer marker Ca199, with gastric cancer progression, the prolonged PFS and OS were at least in part attributable to rebalancing Th1/Th2 and downregulation of CRP, IL-6, and gastric cancer marker Ca199.

Acupuncture and moxibustion is a safe treatment method with few adverse effects, which partly accounts for its wide popularity among patients. The only adverse effect in our study was mild bruising at needling sites and the incidence was 2%. We believe that acupuncture and moxibustion, as valid, safe, convenient “green therapy,” has wide application prospect in the field of tumor treatment rather than only being considered as one of last resorts.

As the first study of the effectiveness of acupuncture and moxibustion on prognosis of advanced gastric cancer, we are aware of its limitations. The first limitation was that the distance between the needling locations of treatment and control groups was only 2 cm. Although the needling depths were not deep enough to obtain arrival of qi in control group, it is possible for sham acupuncture to motivate a little impact to some extent similar to efficacy induced by acupuncture if segmental effect of nerve is taken into account. Furthermore, we are not certain whether the acupoints of acupuncture and moxibustion we chose were optimal. The second limitation was that sham moxibustion was not performed in control group because it is difficult to control the distance between acupoints and nonacupoints in view of high heat conductivity. The third limitation was that the related data detected only at weeks 4 and 8 after the baseline measurements led to lack of long-term data fully accounting for prolonged PFS and OS in treatment group, which, we thought, might be attributable to sustained effect of acupuncture and moxibustion.

In summary, we have provided encouraging preliminary evidence that acupuncture and moxibustion can strikingly improve PFS and OS of advanced gastric cancer patients completing second-line chemotherapy by reversing Th1/Th2 shift and attenuating inflammatory responses. Considering the limitation of small sample size and being single-center, larger-scale, multicenter, randomized controlled trials are required to evaluate conclusively the influence of acupuncture and moxibustion on prognosis of this population.

## Figures and Tables

**Table 1:** The detection primer sequence and amplified range.

Gene primer and probe length (bp)		
T-bet	For: 5′ -GTTCCCATTCCTGTCATTTACT-3′	279
	Rev: 5′ -TCTCCGTCGTTCACCTCAA-3′	
GATA3	For: 5′ -GTAGCTGTAAGGCATGAAGAATG-3′	339
	Rev: 5′-ACTGGTGAACGGTAACACTGATT-3′	
IFN-*γ*	For: 5′-TATTCGGTAACTGACTTG-3 ′	388
	Rev: 5′ -AATCACATAGCCTTGC-3′	
IL-4	For: 5′- CCCCTCTGTTCTTCCTGCTA-3′	368
	Rev: 5′-ACTCTGGTTGCCTTCCTTCA-3′	
GAPDH	For: 5′- GGATTTGGTCGTATTGGG-3′	205
	Rev: 5′-GGAAGATGGTGATGGGATT-3′	

**Table 2:** Baseline characteristics of treatment and control groups.

Parameter	Treatment group	Control group	
	*N* = 81 (%)	*N* = 54 (%)	*P*
Age, *y*			0.879
<60	56 (69.1)	38 (70.4)	
≥60	25 (30.9)	16 (29.6)	
Sex			0.559
Male	50 (61.7)	36 (66.7)	
Female	31 (38.3)	18 (33.3)	
Primary tumor location			0.793
Cardia and fundus	11 (13.6)	5 (9.3)	
Antrum	31 (38.3)	22 (40.7)	
Corpus	36 (44.4)	26 (48.1)	
Whole	3 (3.7)	1 (1.9)	
Primary tumor size			0.603
<6 cm	63 (77.8)	44 (81.5)	
≥6 cm	18 (22.2)	10 (18.5)	
ECOG performance status			0.841
0	32 (39.5)	21 (38.9)	
1	25 (30.9)	19 (35.2)	
2	24 (29.6)	14 (25.9)	
Differentiation			0.675
Well	8 (9.9)	5 (9.3)	
Moderate	45 (55.6)	34 (63.0)	
Poor	28 (34.6)	15 (27.8)	
Metastatic sites per patient			0.888
1-2	38 (46.9)	26 (48.1)	
>2	43 (53.1)	28 (51.9)	
Type of gastric cancer			0.485
Intestinal	42 (51.9)	30 (55.6)	
Diffuse	17 (21.0)	14 (21.9)	
Mixed	22 (27.2)	10 (18.5)	
Extent of disease at study entry			0.818
Locally advanced	8 (9.9)	6 (11.1)	
Metastatic	73 (90.1)	48 (88.9)	

**Table 3:** Effect of acupuncture and moxibustion on IFN-*γ*, IL-4, IL-6, Ca199, and CRP in plasma.

Variables	Group	Baseline	Week 4	Week 8
IFN-*γ* (pg/ml)	Treatment	3.90 ± 1.82	5.88 ± 1.56^*∗*#^	5.81 ± 1.45^*∗*#^
	Control	3.93 ± 0.75	3.53 ± 0.35	3.61 ± 0.26
IL-4 (pg/ml)	Treatment	3.29 ± 1.04	1.82 ± 0.55^*∗*#^	1.65 ± 0.42^*∗*#^
	Control	3.48 ± 1.10	4.15 ± 1.23	4.32 ± 1.56
IL-6 (pg/ml)	Treatment	79.83 ± 41.72	53.29 ± 29.50^*∗*#^	51.18 ± 31.98^*∗*#^
	Control	81.79 ± 25.67	88.66 ±22.65	85.23 ± 26.89
CRP (U/ml)	Treatment	39.73 ± 16.20	28.11 ± 15.63^*∗*#^	30.32 ± 12.53^*∗*#^
	Control	36.52 ± 14.30	44.65 ± 10.68	45.59 ± 12.83
Ca199 (U/ml)	Treatment	60.53 ± 34.21	45.83 ± 21.31^*∗*#^	43.59 ± 25.03^*∗*#^
	Control	58.57 ± 22.57	63.43 ± 22.15	65.89 ± 25.56

Values represent mean ± SD; treatment group (*n* = 81) and control group (*n* = 54). *P* values between baseline measurement and measurements at weeks 4 and 8 in treatment group: ^*∗*^*P* < 0.01; *P* values between measurements in treatment group and measurements in control group at weeks 4 and 8; ^#^*P* < 0.01 (Mann–Whitney *U* test).

## Data Availability

There are no data available for this paper.
